# *Drosophila melanogaster* hosts coevolving with *Pseudomonas entomophila* pathogen show sex-specific patterns of local adaptation

**DOI:** 10.1186/s12862-022-02031-8

**Published:** 2022-06-18

**Authors:** Neetika Ahlawat, Manas Geeta Arun, Komal Maggu, Jigisha, Aparajita Singh, Nagaraj Guru Prasad

**Affiliations:** Department of Biological Sciences, Indian Institute of Science Education and Research Mohali, Sector 81, SAS Nagar, Mohali, 140306 India

**Keywords:** Experimental coevolution, Local adaptation, Sex-specific effects, *Drosophila melanogaster*, *Pseudomonas entomophila*

## Abstract

**Background:**

In spatially structured populations, local adaptation improves organisms’ fitness in their native environment. Hosts and pathogens can rapidly adapt to their local antagonist. Since males and females can differ in their immunocompetence, the patterns of local adaptation can be different between the sexes. However, there is little information about sex differences in local adaptation in host–pathogen systems.

**Results:**

In the current study, we experimentally coevolved four different replicate populations of *Drosophila melanogaster* (host) and *Pseudomonas entomophila* (pathogen) along with appropriate controls. We used the four host–pathogen coevolution populations to investigate the occurrence of local adaptation separately in males and females of the coevolving hosts. We also assessed local adaptation in pathogens. We set up a reciprocal infection experiment where we infected each of the four coevolving hosts with their local pathogen or non-local pathogens from the other three replicate populations. We found that overall, male and female hosts had better survivorship when infected with local pathogens, indicating that they were locally adapted. Interestingly, males were more susceptible to non-local pathogens compared to females. In addition, we found no fecundity cost in females infected with either local or non-local pathogens. We found no evidence of local adaptation among the pathogens.

**Conclusion:**

Our study showed sex-specific adaptation in the coevolving hosts where female hosts had a broader response against allopatric coevolving pathogens with no cost in fecundity. Thus, our results might suggest a novel mechanism that can maintain variation in susceptibility in spatially structured populations.

**Supplementary Information:**

The online version contains supplementary material available at 10.1186/s12862-022-02031-8.

## Background

Populations are said to be “locally adapted” if, as a consequence of spatially varying selection and strong genotype-environment interactions, they evolve characters that improve their fitness in their local environment, irrespective of consequences in foreign environments [1]. Reciprocal transplantation experiments, where the fitness of populations is measured in local versus foreign environments, are a powerful method of investigating local adaptation, and have been used to investigate patterns of local adaptation in a wide range of taxa [2–4]. Traditionally, theoretical and empirical studies investigating local adaptation have overlooked the potential for sex-specific patterns of local adaption. However, there is emerging consensus that patterns of local adaptation need to be investigated in the context of sex-specific selection and sex difference [5–9].

A useful model system for investigating local adaptation is host–pathogen coevolution system. Such antagonistic interactions have the potential to cause rapid evolutionary change in hosts as well as in pathogens. Furthermore, if there are strong genotype (host)–genotype (pathogen) interactions, these processes can lead to patterns of local adaptation where hosts or pathogens evolve characters that are specific to their local antagonist. Analogous to the classical reciprocal transplant experiments, reciprocal cross-infection experiments can be employed to detect potential local adaptation by measuring the fitness of the coevolving hosts and pathogens against their antagonist from local or non-local populations [1–10]. Further, detecting local adaptation in such systems might be difficult as adaptation in one antagonist can be masked by counter-adaptation of the other antagonist. Which of the two antagonists is locally adapted depends on the inflow of new genetic variation through mechanisms like mutation [11]. When local adaptation patterns are observed in one of the two antagonists, then that antagonist is considered to be ahead in the coevolutionary process [11]. Classically, pathogens, due to shorter generation times and larger population sizes, are typically predicted to be locally adapted, and thus ahead in the coevolutionary process [12–14]. However, hosts also generate novel genetic variation by sexual reproduction and dispersal, so the precise dynamics of local adaptation depends upon the natural history of the system [11–15].

Several empirical studies have investigated patterns of local adaptation using various host–pathogen coevolution systems and have found mixed results. For instance, the coevolutionary studies using bacteria-phage system [16], and *Caenorhabditis elegans* (nematode)-*Serratia marcescens* (bacteria) system [17] have shown that coevolving pathogens were more infectious to their local hosts compared to the non-local hosts. These studies showed the presence of local adaptation across coevolving pathogens. In contrast to this, there is a possibility for the antagonists to show no local adaptation or local maladaptation [18]. In such a scenario, the evolved immunity in a host or evolved virulence in a pathogen would be the same or higher against their non-local antagonist compared to their local antagonist. However, in a set of isolated populations, where a host and a pathogen closely interact with each other, it is likely that some populations show patterns of local adaptation while others do not. This creates a mosaic like pattern between different coevolving populations [19, 20]. For instance, studies investigating coevolution using *C. elegans* (nematode)-*Bacillus thuringiensis* (bacteria) [12] and *Tribolium castaneum* (red flour beetle)-*Nosema whitei* (microsporidin parasite) [21] host–pathogen coevolution system had observed that populations were locally adapted against their local parasites while others did not.

Local adaptation studies on host–pathogen systems have typically used bacterial or nematode hosts. These studies provide insights into the consequences of coevolutionary interactions. However, such studies do not provide information about the possible sex-specific nature of coevolutionary interactions. Even the study that did involve a dioecious host *Tribolium castaneum* [21] did not attempt to measure patterns of sex-specific adaptation. In fact, their infection experiments were performed in the larval stage, leading to the possibility that sex-specific adaptation could have interfered with inferred patterns of local adaptation. The sexes can be different in their immunocompetence and therefore, sex-specific selection and intersexual genetic correlations should be considered in studies of local adaptation [5–9]. Immunocompetence is sexually dimorphic in a wide range of taxa [22–24]. Theories that attempt to explain this pattern generally invoke sex-specific selection over immunocompetence and/or its interactions with reproduction, leading to sex-specific fitness optima [25–28]. Consistent with this idea, several empirical studies have reported evidence of sexually antagonistic and/or sex-specific selection over immunocompetence traits [29–35] as well as interactions between reproduction and immunocompetence [36–40]. However, none of these studies have measured sex-specific responses in host–pathogen coevolution systems. Therefore, as populations of hosts coevolve with their respective local pathogens, it is plausible that males and females evolve in distinct ways leading to sex differences in the degree of local adaptation.

In this study we used established replicate experimental coevolution systems between *Drosophila melanogaster* and *Pseudomonas entomophila* [41] to investigate patterns of sex-specific local adaptation. *D. melanogaster* is an excellent host to investigate sex-specific effects because a significant fraction of studies investigating sex difference [42], sexual antagonism [30–32] as well as reproduction-immunity interactions [37, 38, 40] have used *D. melanogaster* as the model system. *P. entomophila* is a gram-negative bacterium, isolated from wild *D. melanogaster* [43]. Infection by *P. entomophila* is lethal to *D. melanogaster* hosts and has been shown to mediate interactions between reproduction and immunocompetence [37, 44] making it an ideal pathogen to investigate patterns of sex-specific adaptation. Furthermore, a host–pathogen coevolutionary study using *D. melanogaster* and *P. entomophila* has reported evidence that in their system, both hosts and pathogens had evolved increased post-infection survivorship and host-killing ability respectively [41]. Additionally, the coevolving hosts were shown to have evolved higher post-infection survivorship relative to hosts evolving against a static pathogen suggesting that the host–pathogen coevolutionary process had led to a distinct outcome compared to one-sided host adaptation. Here, we used four independent replicate *D. melanogaster*-*P. entomophila* experimental coevolution systems set up by the study mentioned above [41] and performed full-factorial cross-infection experiments. To investigate patterns of local adaptation, and whether these patterns exhibited sex differences, we measured the survivorship of male and female hosts from each independent replicate after infecting them with either their respective local pathogen (“sympatric” combinations) or the three other non-local pathogens (“allopatric” combinations). Higher host survivorship in the “sympatric” combinations would indicate local adaptation by the host, while the opposite result would indicate local adaptation in pathogens.

## Results

Our experimental set up consisted of four independent replicate experimental coevolution systems between *Drosophila melanogaster* (host) and *Pseudomonas entomophila* (pathogen). Within each replicate population (Coev 1–4), 200 males and 200 females were infected by the coevolving *P. entomophila* pathogen of that replicate population every generation. Hosts for the next generation were collected from the eggs laid by the flies surviving to 96 h post infection. Flies that died within 24–48 h post infection were stored at 4°C, and were used to isolate the coevolving pathogen for the next generation. Due to experimental contingencies, from the 5th coevolution cycle onwards, fresh coevolving pathogens were isolated once every two generations. See the Materials and Methods section for details on population maintenance.

After 19 cycles of coevolution, we performed the local adaptation experiment. We measured patterns of local adaptation across coevolving hosts and pathogens against their sympatric as well as allopatric antagonist. Hosts from each of the four Coev (1, 2, 3 and 4) populations were infected with the coevolving pathogens (B1Pe or Pe1; B2Pe or Pe 2; B3Pe or Pe 3; B4Pe or Pe 4) from the same population (sympatric combination) or with the pathogens from the other three Coev populations (allopatric combination). Subsequently, we monitored the survivorship of the hosts for 120 h post infection, as well as the fecundity of the females. See the Materials and Methods section for details on experimental protocols and statistical analysis.

### Higher host survivorship against sympatric pathogens

Signature of local adaptation in the host–pathogen coevolution system appears when, either (1) the host exhibits higher survivorship against sympatric than allopatric pathogens, or (2) the pathogen exhibits higher host killing ability against sympatric than allopatric hosts.

We note that causing mortality may not directly indicate the fitness of the pathogen. One possibility is that an increase in fitness would lead to better bacterial growth, and that more bacterial cells can lead to higher lethality. But conversely, evolution can also decrease pathogen lethality, as it often allows for better transmission and thus better fitness. However, in our study only those pathogens that caused fly mortality were chosen to infect the flies of the next generation. Thus, inducing mortality is a pre-requisite for transmission to the next generation in our selection regime.

**Fig. 1:**
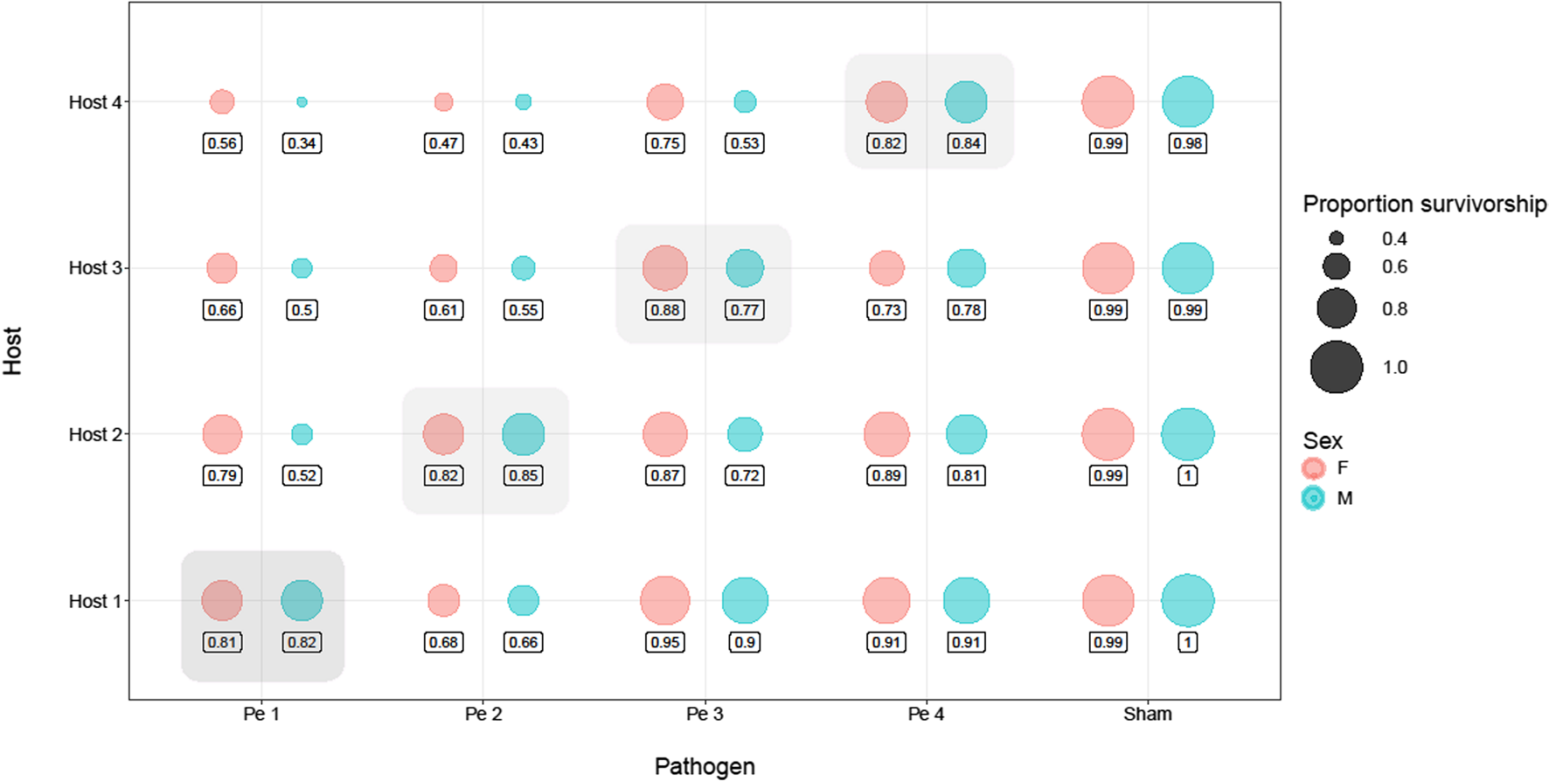
Plot showing the percentage of male and female hosts that were alive after 120 h of infection against sympatric or allopatric pathogens. The pink bubbles represent the proportion survivorship of female hosts (represented as ‘F’), while the blue bubbles represent the proportion survivorship of male hosts (represented as ‘M’)

Results from the survivorship assay are summarized in Fig. 1, which provides a global overview of the surviving proportion of male and female hosts against sympatric and allopatric coevolving pathogens, after 120 h of exposure.

**Fig. 2:**
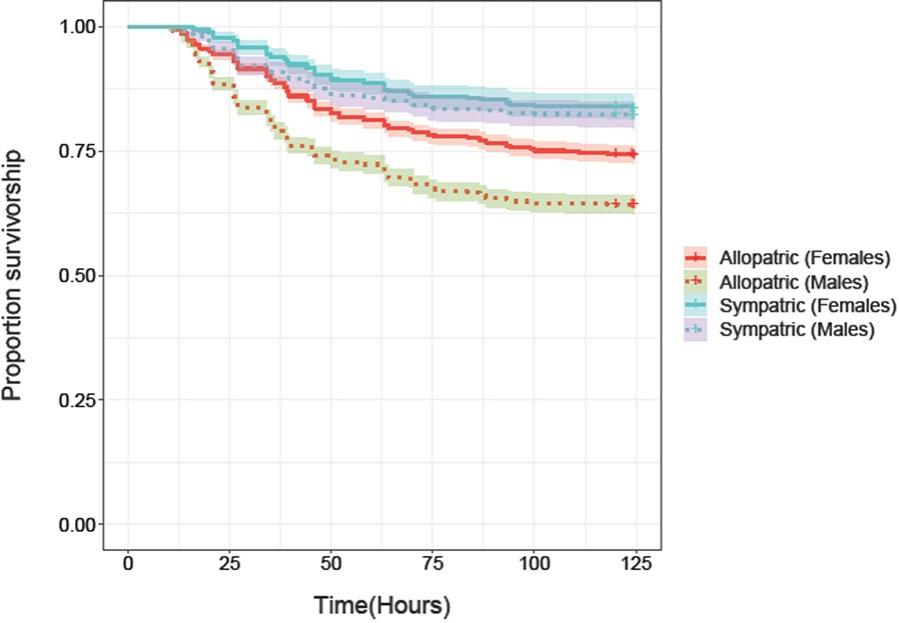
Survivorship of coevolving hosts post infection with sympatric or allopatric pathogens. Solid lines represent survivorship of female hosts while dotted lines represent survivorship of male hosts. Blue lines represent survivorship of individuals when infected with sympatric coevolving pathogens while red lines represent survivorship of individuals when infected with allopatric pathogens. The shading represents confidence intervals (95%)

We fit a Cox proportional hazards model for survivorship of coevolving hosts against sympatric and allopatric pathogens. Overall, we found that male and female coevolving hosts survived better against their sympatric pathogens relative to when infected with allopatric pathogens (Fig. 2). Hazard rate of the coevolving hosts from sympatric combinations was smaller (which indicated better survivorship) than that from allopatric combinations, which was constrained to be 1 in the model (Table 1). We also found a significant effect of sex (with females having higher survivorship overall) and its interaction with the type of pathogen combination i.e. sympatric or allopatric (Table 1). We observed that while male and female coevolving hosts survived better against their sympatric pathogens compared to allopatric pathogens, the magnitude of this difference was considerably higher in males than in females (Fig. 2, Table 1).

**Table 1:** The output of Cox proportional hazards models for coevolving hosts when infected with sympatric or allopatric pathogens

Summary of Cox proportional hazards model			
Fixed coefficients	Hazard ratio	Lower CL	Upper CL
TypeSympatric	0.7016	**0.5150**	**0.9557**
SexMale	1.6042	**1.4548**	**1.7689**
TypeSympatric:SexMale	0.7038	**0.5514**	**0.8983**

Random effects			
Group	Variance		
Replicate	0.0001		
Replicate/type	0.0001		
Replicate/sex	< 0.0001		
Replicate/sex/type	< 0.0001		

Hazard rates are expressed relative to the hazard rates of the default level of each fixed factor, which are constrained to be 1. The default level for “Type” is Allopatric, while the default level for “Sex” is Females. Lower CL and Upper CL indicate lower and upper bounds of 95% confidence intervals. Confidence intervals that do not contain 1 signify statistical significance and are shown in bold. Higher hazard rates are equivalent to lower survivorship in the hosts

Taken together, these results strongly suggest sex-specific local adaptation in the coevolving hosts, with stronger local adaptation in males compared to females. These results also imply that coevolving pathogens were not locally adapted to their hosts.

**Fig. 3:**
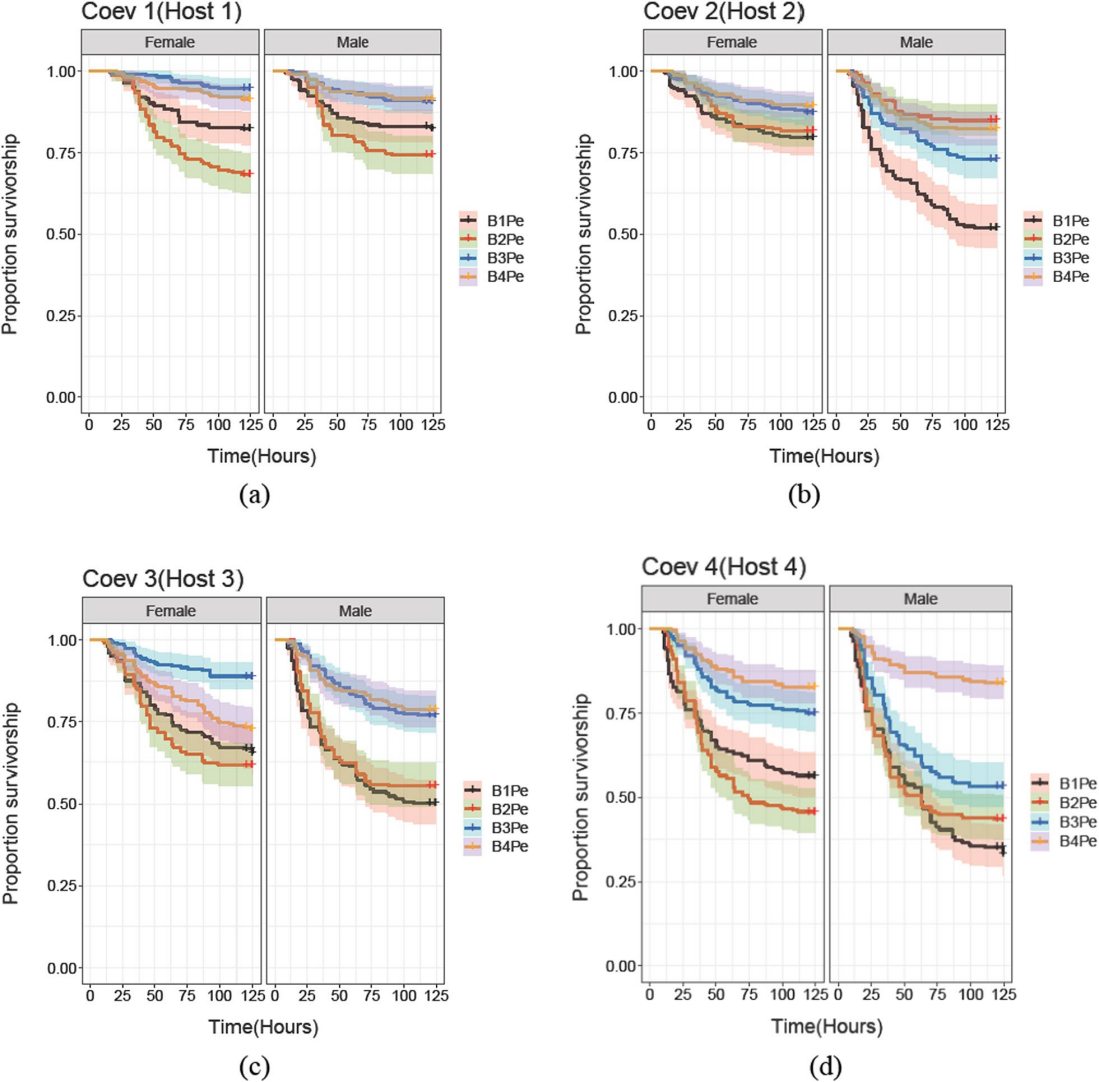
Survivorship curves of Coev 1, Coev 2, Coev 3 and Coev 4 hosts post infection with sympatric and allopatric pathogens. Survival for male and female hosts from each of the four coevolving or Coev populations is plotted in separate graphs with (**a**), (**b**), (**c**) and (**d**) representing Coev 1 hosts, Coev 2 hosts, Coev 3 hosts and Coev 4 hosts, respectively. Within each graph, the left panel represents survivorship of female hosts against sympatric or allopatric pathogens, while the right panel represents survivorship of male hosts against sympatric or allopatric pathogens. Black, red, blue and orange represent survivorship of individuals infected with the coevolving pathogens from B1Pe (Coevolving pathogen for Coev 1 host), B2Pe (Coevolving pathogen for Coev 2 host), B3Pe (Coevolving pathogen for Coev 3 host) and B4Pe (Coevolving pathogen for Coev 4 host), respectively. The shading in each of the survival plot represents confidence intervals (95%)

**Fig. 4:**
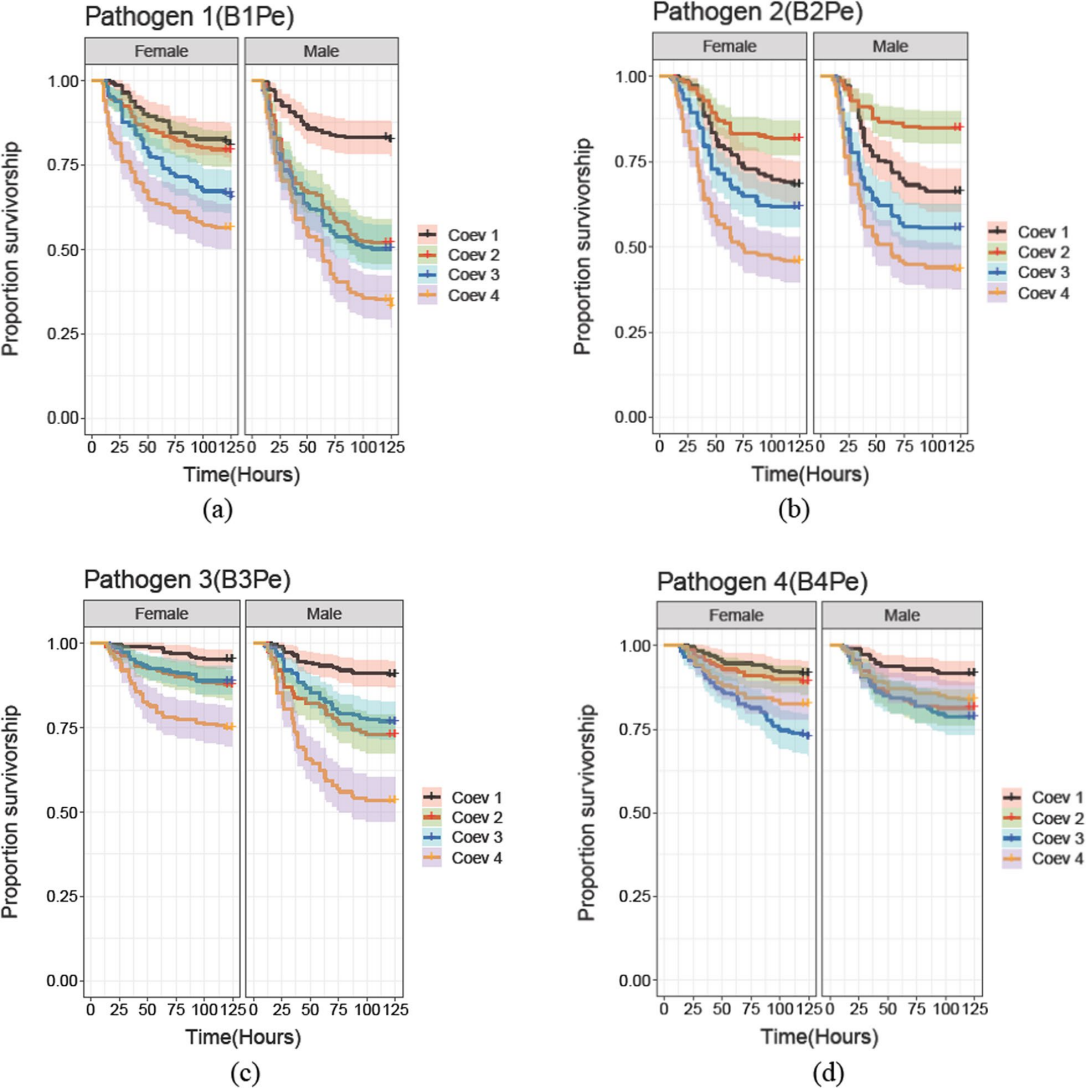
Survivorship curves of sympatric and allopatric hosts when infected with the four coevolving pathogens, (B1Pe, B2Pe, B3Pe and B4Pe). Data for each coevolving pathogen is plotted in separate graphs with (**a**), (**b**), (**c**) and (**d**) representing B1Pe (coevolving pathogen for Coev 1 host), B2Pe (coevolving pathogen for Coev 2 host), B3Pe (coevolving pathogen for Coev 3 host) and B4Pe (coevolving pathogen for Coev 4 host), respectively. Within each graph, the left panel represents survivorship of all four female hosts against each one of the coevolving pathogens, while the right panel represents survivorship of male hosts against each one of the coevolving pathogens. Black, red, blue and orange colours represent survivorship of flies from Coev 1, Coev 2, Coev 3 and Coev 4 populations, respectively

We also observed variability in the evolved traits of hosts and pathogens across the coevolving populations. We observed that B3Pe and B4Pe coevolving pathogens from Coev 3 and Coev 4 populations, in general, caused lower mortality in their coevolving hosts (Coev 3 and Coev 4) relative to coevolving pathogens from other populations (Coev 1 and Coev 2) (Fig. 3, Table 2, Additional file 1: Table S2). In the same way we observed that hosts from populations Coev 3 and Coev 4 exhibited lower survivorship in general, compared to the coevolving hosts from populations Coev 1 and Coev 2 (Fig. 4, Table 3, Additional file 1: Table S3). This meant that in some treatments of host, pathogen and sex (for example, Coev 1 males and females; Coev 2 females), we did not find signals of host local adaptation (Fig. 3, Table 2). In spite of the variability between populations, we do find a global signal indicating sex-specific local adaptation.

**Table 2:** The output of Cox proportional hazards models for different Coev hosts post infection with their sympatric and allopatric pathogens

Summary of Cox proportional hazards model			
COEV 1 (HOST 1)			
Fixed coefficients	Hazard ratio	Lower CL	Upper CL
*Female*			
TypeSympatric	1.2081	0.8381	1.7415
*Random effects*			
*Group*	*Variance*		
Replicate/Type	< 0.0001		
Replicate	< 0.0001		
*Male*			
TypeSympatric	1.2291	0.8479	1.7817
*Random effects*			
*Group*	*Variance*		
Replicate/Type	0.00016		
Replicate	0.053		

COEV 2 (HOST 2)			
Fixed coefficients	Hazard ratio	Lower CL	Upper CL
*Female*			
TypeSympatric	1.2803	0.8879	1.8479
*Random effects*			
*Group*	*Variance*		
Replicate/Type	0.00039		
Replicate	0.0599		
*Male*			
TypeSympatric	0.4440	**0.3018**	**0.6532**
*Random effects*			
*Group*	*Variance*		
Replicate/Type	0.0129		
Replicate	0.0324		

COEV 3 (HOST 3)			
Fixed coefficients	Hazard ratio	Lower CL	Upper CL
*Female*			
TypeSympatric	0.29162	**0.1926**	**0.4415**
*Random effects*			
*Group*	*Variance*		
Replicate/Type	0.00039		
Replicate	0.07937		
*Male*			
TypeSympatric	0.51019	**0.3780**	**0.6885**
*Random effects*			
*Group*	*Variance*		
Replicate/Type	0.00040		
Replicate	0.1387		

COEV 4 (HOST 4)			
Fixed coefficients	Hazard ratio	Lower CL	Upper CL
*Female*			
TypeSympatric	0.3504	**0.2504**	**0.4899**
*Random effects*			
*Group*	*Variance*		
Replicate/Type	0.0004		
Replicate	0.0967		
*Male*			
TypeSympatric	0.19985	**0.1416**	**0.28203**
*Random effects*			
*Group*	*Variance*		
Replicate/Type	0.00049		
Replicate	0.2867		

Hazard rates are expressed relative to the hazard rates of the default level of the fixed factor, which is constrained to be 1. The default level for “Pathogen Type” is Allopatric treatment. Lower CL and Upper CL indicate lower and upper bounds of 95% confidence intervals. Confidence intervals that do not contain 1 signify statistical significance and are shown in bold. Higher hazard rates are equivalent to lower survivorship in the hosts

**Table 3:** The output of Cox proportional hazards models for coevolving pathogens from different populations when they infect their sympatric as well as allopatric hosts

Summary of Cox proportional hazards model			
PATHOGEN 1 (B1Pe)			
Fixed coefficients	Hazard ratio	Lower CL	Upper CL
*Female*			
TypeSympatric	0.5014	**0.3601**	**0.6981**
*Random effects*			
*Group*	*Variance*		
Replicate/Type	0.00018		
Replicate	0.00039		
*Male*			
TypeSympatric	0.2307	**0.1654**	**0.3217**
*Random effects*			
*Group*	*Variance*		
Replicate/Type	0.0004		
Replicate	0.1322		

PATHOGEN 2 (B2Pe)			
Fixed coefficients	Hazard ratio	Lower CL	Upper CL
*Female*			
TypeSympatric	0.37262	**0.2679**	**0.5181**
*Random effects*			
*Group*	*Variance*		
Replicate/Type	0.0004		
Replicate	0.00039		
*Male*			
TypeSympatric	0.2786	**0.1468**	**0.5288**
*Random effects*			
*Group*	*Variance*		
Replicate/Type	0.3316		
Replicate	0.1584		

PATHOGEN 3 (B3Pe)			
Fixed coefficients	Hazard ratio	Lower CL	Upper CL
*Female*			
TypeSympatric	0.7746	0.4986	1.203
*Random effects*			
*Group*	*Variance*		
Replicate/Type	< 0.0001		
Replicate	< 0.0001		
*Male*			
TypeSympatric	0.788	0.5793	1.071
*Random effects*			
*Group*	*Variance*		
Replicate/Type	< 0.0001		
Replicate	< 0.0001		

PATHOGEN 4 (B4Pe)			
Fixed coefficients	Hazard ratio	Lower CL	Upper CL
*Female*			
TypeSympatric	1.1552	0.7992	1.669
*Random effects*			
*Group*	*Variance*		
Replicate/Type	0.00039		
Replicate	0.00012		
*Male*			
TypeSympatric	0.9983	0.6847	1.4554
*Random effects*			
*Group*	*Variance*		
Replicate/Type	< 0.0001		
Replicate	< 0.0001		

Hazard rates are expressed relative to the hazard rates of the default level of the fixed factor, which is constrained to be 1. The default level for “Host Type” is Allopatric treatment. Lower CL and Upper CL indicate lower and upper bounds of 95% confidence intervals. Confidence intervals that do not contain 1 signify statistical significance and are shown in bold. Higher hazard rates are equivalent to lower survivorship in the hosts

### No difference in mean fecundity of females post infection with sympatric or allopatric pathogens

**Fig. 5:**
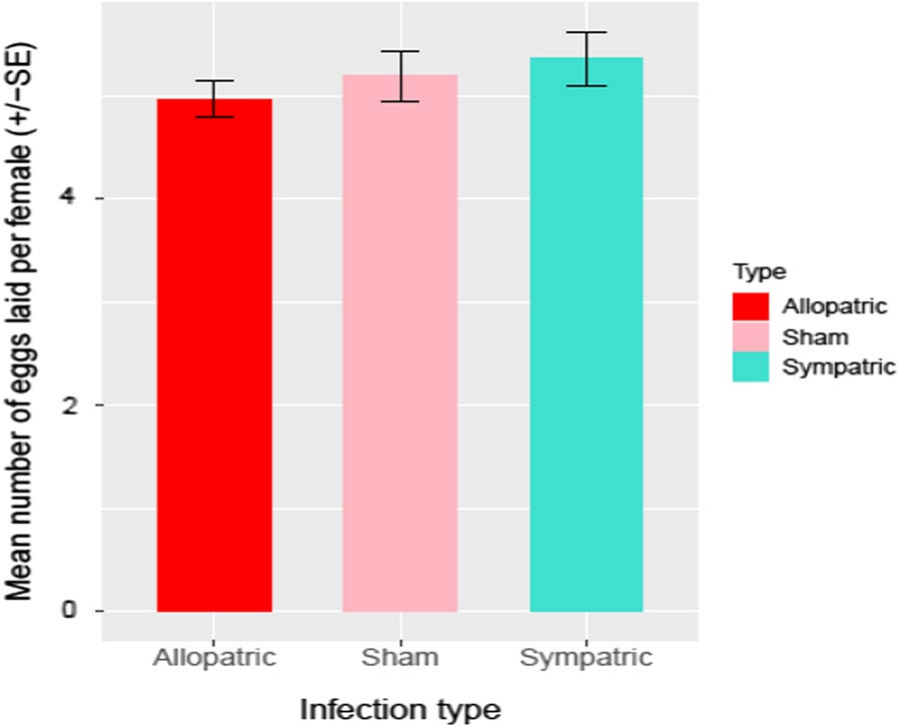
Fecundity of female coevolving hosts, post infection with sympatric or allopatric pathogens or sham infection. The red bar represents fecundity of female hosts post infection with allopatric pathogens while the blue bar represents fecundity of female hosts post infection with sympatric pathogens. The pink bar respresents fecundity of female hosts when sham infected

Mean number of eggs laid per female post infection was used as the unit of analysis. While we observed that the mean fecundity of females when infected with their sympatric pathogens was higher than when infected with allopatric pathogens (Fig. 5), this difference was not statistically significant (Table 4).

**Table 4:** Summary of mixed model anova for fecundity of female coevolving hosts post infection by sympatric or allopatric pathogens

Fixed coefficients	Sum sq	Mean sq	Num Df	Den Df	F value	P(> F)
Type	7.355	3.6777	2	274.3	0.8335	0.4356

Random effects	npar	logLik	ARC	LRT	Df	P(> chisq)
Replicate	5	− 607.79	1225.6	1.557	1	0.212
Replicate:Type	5	− 607.01	1224.0	0	1	1

‘Type’ represents Coev hosts’ combinations with sympatric and allopatric coevolving pathogens. Host–pathogen infection type was considered as fixed factor while experimental replicate was considered as random factor

We also analysed mean fecundity of female hosts from each Coev population separately. Across each coevolving female host, we observed no fecundity difference in females infected with sympatric or allopatric pathogens (Additional file 1: Fig. S1(a-d)). Coevolving hosts from populations Coev 2, Coev 3 and Coev 4 showed a similar trend across their local and non-local pathogens (Additional file 1: Fig. S1(b-d)). However, coevolving host from population Coev 1 had higher fecundity when infected with its local i.e. sympatric pathogen (B1Pe) compared to when infected with allopatric pathogens (Additional file 1: Fig. S1a).

## Discussion

We conducted full factorial cross-infection experiments using replicated experimental coevolution systems of *D. melanogaster* (host) and *P. entomophila* (pathogen)*.* We found that, on average, both male and female hosts had higher survivorship when infected with their respective sympatric coevolving pathogen, compared to when they were infected with allopatric coevolving pathogens, suggesting hosts (and not pathogens) were locally adapted to their sympatric antagonist. Additionally, the drop in host survivorship when infected with allopatric versus sympatric pathogens, was considerably more drastic in males compared to females. This is among the first reports of sex-specific local adaptation in any host–pathogen coevolutionary system. Furthermore, we could detect no differences in female host fecundity post infection with sympatric or allopatric pathogens. Overall, we also found that there was variability among the Coev populations (Coev 1–4) for host survivorship, and for the pathogen’s ability to induce mortality in hosts.

An important caveat of our study is that in each of our four populations of host–pathogen coevolution systems, two consecutive generations of hosts were infected by the same isolate of the pathogen. Subsequently, a fresh isolate of the pathogen was prepared by culturing the pathogen from flies that died in the first two days after being infected in the second generation. This isolate of the pathogen was then used to infect the next two consecutive host generations, and so on. This approach was necessitated by initial rapid evolution of pathogen virulence, leading to a higher than optimal host mortality. However, this meant that our experimental design was actually a hybrid of single-sided host adaptation and host–pathogen coevolution. Nevertheless, both hosts and pathogens rapidly evolved greater resistance and virulence respectively within 20 coevolution cycles [41]. Furthermore, each of the four coevolving host populations had their own specific pathogen population (B1Pe, B2Pe, B3Pe and B4Pe), they were coevolving with. Each of these four host–pathogen coevolution systems was always maintained independently; i.e. the hosts from Coev 1 population never encountered pathogens from Coev 4 i.e. B4Pe population, and so on. Therefore, our experimental host–pathogen coevolution systems are well-suited to address questions of local adaptation.

Although our results clearly show an overall pattern of host local adaptation, when the survivorship results were examined individually across each of the Coev hosts, some combinations showed local adaptation while some did not. In our experimental design, while variation in host survivorship is a function of sex, type of host–pathogen interaction (sympatric versus allopatric) and their interaction, it can also be affected by variation in absolute (as opposed to antagonist-specific) pathogen virulence and host resistance across replicates. We modeled these absolute effects associated with host identity and pathogen identity as random intercepts in our Cox proportional hazards model, and detected fairly large corresponding variance estimates (0.2366 for hosts, and 0.2578 for pathogens). Therefore, it is not entirely surprising that in certain replicates, these strong effects associated with absolute host resistance and pathogen resistance masked local adaptation patterns. For instance, B3Pe and B4Pe pathogens from Coev 3 and Coev 4 populations respectively, evolved at a slower pace, i.e. they killed their own hosts as well as non-local hosts at a lower rate than coevolving pathogens from Coev 1 and Coev 2 populations [45]. This results in hosts from Coev 1 and Coev 2 having higher survivorship against pathogens from Coev 3 and Coev 4, relative to when infected with their respective sympatric pathogens (Fig. 3). However, interestingly, Coev 1 hosts had higher survivorship against their sympatric pathogen relative to pathogen from Coev 2 i.e. B2Pe, and similarly, Coev 2 hosts had higher survivorship against their sympatric pathogen relative to the Coev 1 pathogen i.e. B1Pe (Figs. 3 and 4). Coev 4 hosts clearly had the highest survivorship against their sympatric pathogen (Fig. 3a). Coev 3 females had the highest survivorship against their sympatric pathogen, while Coev 3 males had comparable survivorship against Coev 3 and Coev 4 pathogens, but considerably higher than against Coev 1 and Coev 2 pathogens (Fig. 3c). Viewed together, it is reasonable to interpret these patterns as a combined effect of local host adaptation and variation in absolute pathogen virulence and/or host resistance.

Studies investigating patterns of local adaptation have, typically, either measured the fitness of only one of the sexes or have averaged over the fitness of males and females [7]. However, incorporating sex-specific effects and sex-differences in paradigms of local adaptation can lead to novel insights [5–9]. A few empirical studies have explicitly tested for sex-specific local adaptation patterns. For example, a study measured fitness components of two hermaphroditic ragweed *Abrosia artimisiifolia* populations in their respective native versus foreign environments. The study showed that plants from two different geographical regions outperformed foreigner plants in their respective local regions. Residents in each of the local regions showed higher seed production (a measure of female fitness) and increased height of flowers (and therefore, pollen dispersal, a measure of male fitness) respectively, relative to the other foreign populations [46]. Similarly, sex-specific local adaptation has also been inferred in dewlaps (for signal communication), a sex-limited and shared morphological traits primarily expressed in male *Anolis sagrei* [47] as well as with respect to cryptic colouration in rock dragon lizards *Ctenophorus decresii* [48]. On the other hand, another study found that patterns of local adaptation in populations of two *Silene* sister species were comparable between males and females [49]. It is important to note that none of these studies have measured sex-specific effects of local adaptation in host-pathogen coevolution systems. In this context, our finding that different replicate populations of *D. melanogaster* (host)*-P. entomophila* (pathogen) coevolution systems showed sex-specific local adaptation against their pathogenic antagonist is novel and important. In our full factorial cross-infection experiments, we found a strong interaction between sex and the type of host–pathogen interaction (sympatric versus allopatric). When infected with their sympatric pathogens, male and female hosts from the coevolving populations had comparable survivorship. However, when infected with allopatric pathogens, male survivorship reduced to a considerably larger extent than female survivorship. This suggests that, while coevolving with their respective sympatric pathogens, females had evolved a broader response that was to a great extent also effective against allopatric pathogens. Males, on the other hand, appeared to have evolved a response that was much more tailored to their respective sympatric pathogens. Svensson et al. [7] had argued that sex-specific patterns of local adaptation could arise if fitness peaks for phenotypes shift in different ways (both in terms of magnitude and direction) for males and females between environments (see Fig. 6 in [7]). However, they had assumed that the fitness peaks of both males and females had identical forms. Our results may suggest that if the female fitness peaks are broader than male peaks, patterns of sex-specific local adaptation can arise, even when male and female fitness optima coincide in each environment.

**Fig. 6:**
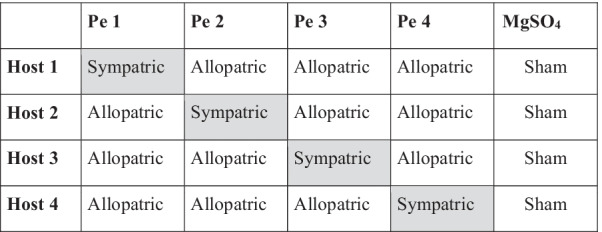
Matrix showing host–pathogen treatments for local adaptation experiment. Host combinations are shown across rows and pathogen combinations are shown across columns. The shaded area indicates sympatric (local antagonist) combinations rest others are allopatric (foreign antagonist) combinations

The mechanistic basis of why females would evolve a broader evolved response to their coevolving pathogens than males, would require further investigation. One possibility is that coevolving males and females might invest differently in maintaining immunity [39] or might employ distinct evolved strategies (possibly by employing different aspects of the immune response) to maximise their fitness. This is a definite possibility in our system given that surviving bacterial infection was essential for females to have non-zero fitness, while males had other avenues of siring progeny (for example, by channeling greater investment in their ejaculate) even without investing in clearing the infection per se. There is also robust evidence for reproduction-immunity interactions [37, 38, 40] as well as sexual dimorphism [42], sex-specificity and sexual antagonism [30–32] over immunocompetence in *D. melanogaster*. Apart from *quantitative* sex-differences in various parameters of the immune response [22–24], particularly in vertebrates, male and female immune responses are also known to be *qualitatively* different [28, 50–53], suggesting that females might invest more in pathogen detection, while males in targeting those pathogens. We hypothesise that these quantitative and/or qualitative sex-differences in immunocompetence could have driven the evolution of sex-specific patterns of local adaptation in our study.

An interesting insight from our results is that patterns of sex-specific local adaptation could drive population level sex-differences in immunocompetence. In our cross-infection experiments, we found sex-differences only in the allopatric treatments, where males had poorer survivorship relative to females. There were no sex differences in the sympatric treatments. Our study can potentially provide insights into the role of dispersal in maintaining sex-differences in immunocompetence in a spatially-structured population where the male and female hosts coevolve against spatially-limited local pathogens. Reduced expression of immune traits as a result of dispersal to the foreign environment has been characterized in *Chorthippus biguttulus* grasshopper as well as in amphibians, *Rhinella marina* (cane toad) [54]. However, these studies lack the understanding of sex-specific effects of dispersal on immunocompetence. As a result of dispersal, if females acquire broader evolved responses compared to males, the resident or local males and females would have comparable immunocompetence but immigrant males would have relatively poor immunocompetence compared to immigrant females. Therefore, sex-differences in immunocompetence can in principle be a consequence of the presence of immigrant males and females. This might suggest that sex-specific difference in traits is analogous to local adaptation where males and females represent different environment to the pathogen.

In host–pathogen coevolutionary systems, pathogens are typically expected to be locally adapted, owing to their large effective population sizes [12–14]. The experimental evidence for this hypothesis is mixed. Some studies found that coevolving pathogens have greater fitness against their local hosts [16, 17]. Our findings that coevolving pathogens caused less mortality to their sympatric hosts are consistent with other studies that have reported local maladaptation in pathogens [55, 56]. This could potentially be a consequence of a combination of several technical aspects of our experimental set up. First, as described above, to reduce excessive mortality in the hosts, our coevolution design alternated between host–pathogen coevolution and single-sided host adaptation. Second, after isolating pathogens from dead flies, fresh cultures were set up using approximately 10 colonies. Therefore, the population sizes of the coevolving pathogens went down to 10 individuals in our design. Lastly, the coevolving pathogens also spent considerable time while growing in LB, where they were presumably under selection for faster growth in that medium [45]. All these factors could have slowed down pathogen adaptation to their respective coevolving hosts, thereby yielding no patterns of local adaptation in our experiments.

We also expected that the allopatric pathogens may have effects on resource investment in other traits in the coevolving hosts. We hypothesized that alleles enhancing host adaptation against local pathogens might affect hosts’ fitness in response to infection by allopatric pathogens [57]. We thus measured the physiological costs associated with survivorship against sympatric or allopatric pathogens as the number of eggs laid by females post infection. We did not find any difference in the fecundity of sham infected females, females infected with sympatric pathogens or females infected with allopatric pathogens. Thus, better survivorship of female hosts against allopatric pathogens did not carry fecundity cost of infection with either sympatric or allopatric pathogens. Analogous to our study, spider mites evolving on different plant species were found to incur no cost of adaptation [58]. However, studies measuring the costs associated with dispersal or adaptation in a non-local environment are rare and results are ambiguous [58, 59]. Observing no fecundity cost against allopatric pathogens indicates that either generating an immune response is probably cheap or that the likelihood of resemblance between sympatric and allopatric pathogens is fairly high. While we did not measure any of the life-history traits in the male hosts, we speculate that one of the reasons for the relatively high susceptibility of males against allopatric pathogens, could be their reproductive investment. Hence, quantification of male reproductive investment in the face of challenge from allopatric vs sympatric pathogens might be very instructive.

## Conclusion

In conclusion, using experimental coevolution between *D. melanogaster* and *P. entomophila* we found that hosts, and not pathogens, exhibited adaptation towards their local pathogens. We also found evidence of sex-specific local adaptation, with females evolving a broader response that was reasonably effective against non-local pathogens as well, and males evolving a response more specific to their local pathogen. Lastly, our results also suggest that sex-specific local adaptation can lead to sex-differences in average immune phenotypes in spatially structured populations. In addition, we did not observe any fecundity cost of the increased female survival against sympatric or allopatric pathogens. These results suggest that studies of coevolution systems involving diecious hosts need to account for the possible sex-specific patterns of coevolution.

## Methods

The current study used a set of four populations of *Drosophila melanogaster* that have been coevolving with a Gram-negative bacterial pathogen *Pseudomonas entomophila* which carried ampicillin and rifampicin resistant genes. These four populations are a subset of a set of 16 replicate populations reported by Ahlawat et al. [45]. In the other 12 populations of Ahlawat et al. [45] the hosts and pathogens were not allowed to coevolve. Hence, in the current study we have used only the four coevolving populations and not the other 12 populations.

The complete protocol for experimental evolution set-up can be found in the Additional file 1. Briefly, we derived the four coevolution populations (called Coev 1–4) from four replicate laboratory adapted populations of *Drosophila melanogaster* known as BRB 1–4 (Blue Ridge Baseline) (see Additional file 1). Coev 1 was derived from BRB 1, Coev 2 was derived from BRB 2 and so on. The Coev populations were maintained on a 16 day discrete generation cycle, 25 ℃, 50–60% RH on standard banana-jaggery food. On day 12 post egg collection, when flies were roughly 2–3 days old as adults, 200 males and 200 females (20 males and 20 females from each of the 10 culture vials) from each population were infected with a needle dipped in a suspension (OD_600_ 0.4) of coevolving *P. entomophila* pathogen. Flies start dying after about 12 h of infection with peak mortality arrives 24 h after infection. By 96 h after infection, the mortality plateaus. Therefore, we recorded host mortality until 96 h after infection, at which time, about 200 flies (~ 50%) would survive the infection. After 96 h of infection (day 16 post egg collection), the surviving flies would be provided with a fresh food plate for 18 h for oviposition. These eggs were collected and dispensed into 10 food vials (90 mm length × 25 mm diameter) containing 6–7 ml of standard banana jaggery food at a density of 70 eggs per vial. These vials were then incubated at standard laboratory conditions to start the next generation.

Within 24–48 h post infection when flies were dying, we collected 10–15 dead flies per sex. Later, we used these dead flies to extract the bacteria to infect the next generation coevolving host. Out of these dead flies, five flies were randomly picked and were transferred into micro-centrifuge tubes after surface-sterilization. These flies were then crushed in sterile 10 mM MgSO_4_ and the fly sample was diluted 3–4 times (dilution ratio 1:1000 to 1:10,000). We plated this diluted sample on LB agar plates containing ampicillin which were then incubated at 27°C. We randomly picked 11–12 colonies from different regions and used an overnight culture to infect the flies. It is important to note that for each of the four coevolving hosts (Coev 1, Coev 2, Coev 3 and Coev 4) we had four matched coevolving *Pseudomonas entomophila* pathogens. These four coevolving pathogens are designated as ‘B1Pe’, ‘B2Pe’, ‘B3Pe’ and ‘B4Pe’. The pathogen isolated from dead flies of Coev 1 population was used to infect the next generation host of Coev 1 population only (and not Coev 2, 3 or 4). Similarly, the pathogen isolated from dead flies of Coev 2 population was used to infect the next generation host of Coev 2 population only (and not Coev 1, 3 or 4) and so on. Thus, the host and the pathogen formed a coevolving pair. Therefore, we had four such matched pairs of coevolving host and pathogen (Coev 1 with B1Pe; Coev 2 with B2Pe etc.). These four matched pairs are independent replicate populations of the coevolutionary experimental system. It is also important to note that each of the four matched pairs formed a sympatric pair.

In the initial five generations, as a result of rapid evolution of the coevolving pathogens, we observed increased mortality of the coevolving host each generation. Hence, to provide sufficient time to the host to (co)evolve and to maintain sufficient survivors to contribute to next generation, we started to infect two consecutive host generations with one generation of pathogen, after the 5th coevolution cycle. In other words, after two generations of host evolution, a new coevolution cycle for host and pathogen was proceeded. A fresh sample of coevolving bacteria was isolated from the host only after allowing it to evolve for two generations against the coevolved bacteria from the previous generation. This practice ensured sufficient time for the coevolving host to coevolve with the pathogen.

After 19 cycles of host–pathogen coevolution or approximately 33 cycles of host evolution in response to coevolving pathogen, we conduted the current experiment (see below).

### *Pseudomonas entomophila* pathogen

This pathogen was isolated from wild *Drosophila melanogaster* and causes a significant amount of mortality in the flies [43]. We provided systemic infections to the flies on the lateral region of the thorax, using a fine minuteium sterile needle. All the fly infections were done following the same protocol as mentioned in the Additional file 1.

Results from our trial experiments showed that this pathogen is virulent to the flies and causes around 60% mortality in the flies at a bacterial optical density (OD_600_) of 0.5. This pathogen is preserved at – 80℃ and this preserved stock of *P. entomophila* is referred to as Ancestral Pe. The coevolving pathogen of each replicate of Coev regime are first derived from this preserved stock of *P. entomphila*. Thus, this stock is the ancestor for all the coevolving pathogens.

### Local adaptation experiment

In this experiment we investigated if the coevolving host or pathogen was locally adapted. We assessed two traits in hosts (a) survivorship post infection and (b) fecundity post infection in females.

If a host survives better against infection from its local (sympatric) coevolving pathogen, compared to infection from non-local pathogens, then, it would indicate host local adaptation. For example, if Coev 1 host had higher survivorship against B1Pe as compared to other allopatric (or non-local) pathogens (B2Pe, B3Pe and B4Pe), this would indicate Coev 1 host is locally adapted. Similarly, if the coevolving pathogen causes higher mortality in its local (or sympatric) host (compared to mortality induced in non-local hosts), it would indicate pathogen local adaptation. For example, if B1Pe causes higher mortality in Coev 1 host as compared to allopatric (or non-local) hosts (Coev 2, Coev 3 and Coev 4), it would indicate that B1Pe was locally adapted. Therefore, we measured local adaptation by infecting each of the four Coev hosts individually with their sympatric or allopatric coevolving pathogens (B1Pe, B2Pe, B3Pe and B4Pe). For each of the four hosts, we also ran a sham infected control. This gave us a combination of 4 hosts × 5 treatments (1 sympatric pathogen + 3 allopatric pathogens + 1 sham infection control) with a total of 20 treatments (Fig. 6). Thus, there were 4 sympatric treatments (Coev 1 flies infected with B1Pe, Coev 2 infected with B2Pe and so on) and 12 allopatric treatments (Coev 1 flies infected with B2Pe or B3Pe or B4Pe and so on) (Fig. 6). In the 4 sham-infection control treatments, the experimental flies from each Coev population were injured with a needle dipped in sterile 10 mM MgSO_4_ solution. We use MgSO_4_ solution to prepare bacterial suspension and it confers negligible (0–1%) fly mortality. It is thus used as a control for bacterial infections in the host. This whole experiment was independently repeated on three different days, yielding three independent experimental replicates. Therefore, in total, we infected/sham infected a total of 9000 flies for the experiment—150 flies per treatment (75 males + 75 females) × 20 treatments × 3 experimental replicates.

To ensure observing genetic consequences from our selection treatments separated from potential parental effects, we maintained flies from each Coev (Coev 1, Coev 2, Coev 3 and Coev 4) populations on standard conditions (maintained under uninfected, common garden conditions for one generation—see Additional file 1) for one generation before the assays. For the experiment, eggs were collected from these standardized flies at a density of 70 eggs per vial containing 6–7 ml banana-jaggery food. Forty such vials were collected for each of the Coev populations and these vials were incubated under standard laboratory conditions (mentioned above).

On the 12th day post egg collection, when the flies were roughly 2–3 days old as adults, 75 males and 75 females were randomly chosen for each infection (one sympatric and three allopatric infection treatments for each Coev population) and sham control treatment. These flies were then anesthetized using CO_2_ and infected by pricking the thorax with a needle dipped in bacterial slurry (for detailed protocol, see Additional file 1). Experimental flies of each of the Coev populations were infected with the coevolving pathogens following the respective allopatric and sympatric treatments (Fig. 6). Post infection, the 75 male and 75 female flies from each treatment were transferred to their respective cage and were provided with a fresh food plate. Post-infection mortality was recorded in each of these experimental cages every 3–4 h for the first 48 h, and then every 6–8 h till 120 h.

*Fecundity across sympatric and allopatric populations* We used female flies from the survival experiment (see above) to measure fecundity across each combination of selected flies and pathogen along with sham control. Post infection, a fresh food plate was provided to each cage for 6 h. Plates were provided between 4 pm to 10 pm to account for the fecundity peak that we observe in our flies when switching to the dark part of the light cycle. After 6 h, plates from each cage were replaced with new food plates. These fecundity plates were provided daily to each cage, up until the 120 h time-point. These fecundity plates were labeled as per the day and combination, and stored at – 20°C. Later, these plates were thawed and the eggs were counted. For each cage, we knew the number of females alive at the start of each egg-laying window (see the mortality data collection in the previous experiment). We used the number of females alive at the start of each egg laying window to calculate the number of eggs laid per female in each cage.

### Statistical analysis

All the analyses were done using R.4.0.2 (R Core Team).

*Host resistance and pathogen virulence across sympatric and allopatric combinations* To investigate patterns of local adaptation, we classified the host–pathogen interactions in our experiment into two types:

1. Sympatric—Both host and pathogen were from the same population (Coev 1 host infected with B1Pe, Coev 2 host infected with B2Pe and so on)
2. Allopatric—Host and pathogen were from different populations (eg. Coev 1 host infected with B2Pe, B3Pe or B4Pe; Coev 3 host infected with B1Pe, B2Pe or B4Pe and so on)

We fit a Cox proportional hazards model using the ‘coxme’ package [60] that incorporated the type of interaction (sympatric versus allopatric) and sex as fixed factors. The host population, the pathogen population and the interaction between the two were treated as random. The three experimental replicates were also treated as random.

We used the following model:

Survivorship ~ Type + Sex + Type:Sex + (1 | Host) + (1 | Pathogen) + (1 | Host/Pathogen) + (1 | Replicate).

In addition to the complete model, we also fitted sixteen smaller models. Eight of these used the survivorship of male individuals, and the other eight used the survivorship of female individuals. Among the eight models for each sex, there were four models that investigated patterns of local adaptation for each host separately (that is, four separate models for Coev 1, Coev 2, Coev 3, and Coev 4), along with four models that investigated patterns of local adaptation for each pathogen separately (that is, four separate models for B1Pe, B2Pe, B3Pe, and B4Pe). Each of these sixteen models treated the type of coevolutionary interaction (sympatric vs allopatric) as a fixed factor, while replicate and its interaction with type were treated as random factors, as follows:

Survivorship ~ Type + (1 | Replicate/Type) + (1 | Replicate).

*Host fecundity across sympatric and allopatric combinations* To investigate female fecundity effects in each combination of Host × Pathogen (or sham), we used linear models from ‘lme 4’ [61] and ‘lmerTest’ [62] packages. We analyzed the number of laid eggs as a response variable, modeled as a function of the type of interaction (sympatric versus allopatric) as fixed factor, and the three experimental replicates were treated as random factor. We also measured fecundity of each host when infected with its sympatric or allopatric pathogen (see Additional file 1). Mean and standard error for each treatment was calculated using ‘summarySE()’ function under ‘Rmisc’ [63] package.

Eggs laid per female ~ Type + (1|Replicate) + (1|Replicate:Type).

## Supplementary Information

**Additional file 1: Figure S1.** Mean fecundity of females from each of the coevolving populations post infection by sympatric or allopatric coevolving pathogens, or post sham infection. Error bars represent standard errors. Data for each of the four coevolving host populations is plotted in separate graphs with (a), (b), (c) and (d) representing Coev 1 hosts, Coev 2 hosts, Coev 3 hosts and Coev 4 hosts respectively. H1, H2, H3 and H4 represent Coev 1, Coev 2, Coev 3 and Coev 4, and P1, P2, P3 and P4 represent coevolving pathogens from those populations, i.e., B1Pe (coevolving Pe from block 1), B2Pe (coevolving Pe from block 2), B3Pe (coevolving Pe from block 3) and B4Pe (coevolving Pe from block 4) respectively. Within each graph, pink, grey, light blue, orange and purple colours represent fecundity of female hosts post infection by B1Pe, B2Pe, B3Pe, B4Pe and sham infection respectively. **Table S1.** Summary of mixed model anova for fecundity of females from the four coevolving populations when subjected to five different infections treatments: sham infection or infected with B1Pe, B2Pe, B3Pe or B4Pe. Treatment was considered as a fixed factor while experimental replicate was considered as a random factor. **Table S2.** The output of Cox’s proportional hazards models for different Coev hosts post infection with their sympatric and allopatric pathogens. Hazard rates are expressed relative to the hazard rates of the default level of each fixed factor, which are constrained to be 1. The default level for “Pathogen” is Pe 1, while the default level for “Sex” is Females (F). Lower CI and Upper CI indicate lower and upper bounds of 95% confidence intervals. Confidence intervals that do not contain 1 signify statistical significance and are shown in bold. Higher hazard rates are equivalent to lower survivorship in the hosts. In the table, B1Pe, B2Pe, B3Pe and B4Pe represent coevolving Pe from population Coev 1, Coev 2, Coev 3 and Coev 4. **Table S3.** The output of Cox’s proportional hazards models for each of the coevolving pathogens from different populations when they infect their sympatric as well as allopatric hosts. Hazard rates are expressed relative to the hazard rates of the default level of each fixed factor, which are constrained to be 1. The default level for “Host” is Coev 1, while the default level for “Sex” is Female (F). Lower CI and Upper CI indicate lower and upper bounds of 95% confidence intervals. Confidence intervals that do not contain 1 signify statistical significance and are shown in bold. Higher hazard rates are equivalent to lower survivorship in the hosts.

## Data Availability

Data are available at https://datadryad.org/stash/share/RJea0Rzw79CGnO4Xhhf1dNuTZo29mkL4-pehesTkBQ8.
