# Supplementary material for: Drosophila melanogaster hosts coevolving with Pseudomonas entomophila pathogen show sex-specific patterns of local adaptation
Source: BMC Ecol Evol. 2022 Jun 18;22:77. doi: 10.1186/s12862-022-02031-8 (PMC9206745; doi:10.1186/s12862-022-02031-8)
Supplement: Supplementary file 1 — Additional file 1: Figure S1. Mean fecundity of females from each of the coevolving populations post infection by sympatric or allopatric coevolving pathogens, or post sham infection. Error bars represent standard errors. Data for each of the four coevolving host populations is plotted in separate graphs with (a), (b), (c) and (d) representing Coev 1 hosts, Coev 2 hosts, Coev 3 hosts and Coev 4 hosts respectively. H1, H2, H3 and H4 represent Coev 1, Coev 2, Coev 3 and Coev 4, and P1, P2, P3 and P4 represent coevolving pathogens from those populations, i.e., B1Pe (coevolving Pe from block 1), B2Pe (coevolving Pe from block 2), B3Pe (coevolving Pe from block 3) and B4Pe (coevolving Pe from block 4) respectively. Within each graph, pink, grey, light blue, orange and purple colours represent fecundity of female hosts post infection by B1Pe, B2Pe, B3Pe, B4Pe and sham infection respectively. Table S1. Summary of mixed model anova for fecundity of females from the four coevolving populations when subjected to five different infections treatments: sham infection or infected with B1Pe, B2Pe, B3Pe or B4Pe. Treatment was considered as a fixed factor while experimental replicate was considered as a random factor. Table S2. The output of Cox’s proportional hazards models for different Coev hosts post infection with their sympatric and allopatric pathogens. Hazard rates are expressed relative to the hazard rates of the default level of each fixed factor, which are constrained to be 1. The default level for “Pathogen” is Pe 1, while the default level for “Sex” is Females (F). Lower CI and Upper CI indicate lower and upper bounds of 95% confidence intervals. Confidence intervals that do not contain 1 signify statistical significance and are shown in bold. Higher hazard rates are equivalent to lower survivorship in the hosts. In the table, B1Pe, B2Pe, B3Pe and B4Pe represent coevolving Pe from population Coev 1, Coev 2, Coev 3 and Coev 4. Table S3. The output of Cox’s proportional h [file 12862_2022_2031_MOESM1_ESM.docx]

**Materials and Methods**

**Fly stocks and Bacterial Stocks**

**Bacterial Stocks**:- We used the bacteria *Pseudomonas entomophila* for fly infections. It is a gram negative, rod shaped bacteria, isolated from *Drosophila melanogaster* (Dieppois *et al.*, 2015). The bacterial strain is GFP tagged and carries ampicillin and rifampicin resistant genes. All the bacterial stocks i.e., evolved and non-evolved were stored at -80°C by preparing glycerol stock solutions. The bacteria are cultured in LB medium along with ampicillin (10µl/ml) at 27°C. From the initial set of trial experiments, this pathogen was found to be virulent to the flies and caused around 60% mortality in the flies at an OD_600_ of 0.4.

**Fly Stocks**:- All the fly stocks were derived from the laboratory adapted populations known as BRBs (Blue Ridge Baseline). These populations are maintained on banana-jaggery food (1litre of food contains Banana (205g), barley flour (25g), jaggery (35g), live yeast (36g), agar (12.4g), ethanol (45ml), p-Hydroxymethyl benzoate (2.4g) and water (1800ml)) at standard laboratory conditions of 25°C temperature and 50-60% relative humidity. BRBs are large outbred populations and consist of 5 independent replicates termed as Blocks. Each of the blocks in BRB populations trace their ancestry to a total of 19 isofemale lines and the female flies in these lines were sampled from Blue Ridge Mountains, USA. BRBs have been maintained in our laboratory for >200 generations. Each block (replicate population) of BRB contains ~2800 individuals and is maintained on a 14 day discrete life cycle. For each block, eggs are collected every generation in 40 food (6-7ml banana-jaggery fly food) vials at a density of 70 eggs per vial. Later, on 12^th^ day, when the flies are roughly 2-3 days old as adults, flies from each of the 40 vials were transferred into a large cage provided with a banana-jaggery food plate supplemented with yeast for 48 hours. Later on 14^th^ day, eggs were collected for each of the 5 blocks in 40 food vials for each of the five blocks of BRB.

We used four of these BRB (BRB 1-4) populations to derive four selection regimes for our experimental set-up.

The four selection regimes were:

1. Coevolution (Coev 1-4): (both host and pathogen coevolve)
2. Adaptation (Adapt 1-4): (only host evolves in response to a non-evolving pathogen)
3. Sham control (Co.S 1-4): (infection or injury control)
4. Unhandled control (Co.U 1-4): (untreated control)

In this study we only used populations from the Coev selection regime. However, below, we describe all selection regimes in detail.

**Selection Regimes**

**1. Coev Regime (host - pathogen coevolution):** There were four populations in the Coev selection regime, each labelled as Coev 1, Coev 2, Coev 3 and Coev 4 respectively. For each Coev population, eggs were collected in 10 food vials (90-mm length × 25-mm diameter) at a density of 70 eggs per vial containing 6-7 ml of standard banana-jaggery food. The vials were incubated at 25ºC temperature, 50-60% relative humidity and under 12:12LD cycle. Under these conditions, the flies start eclosing on day 9 post egg collection with peak eclosion on day 10 post egg collection. On day 12 post egg collection, when the flies were 2-3 days old as adults, for each Coev population, 200 males and 200 females (20 males and 20 females from each rearing vial) were randomly chosen and were lightly anesthetised using CO_2_. They were infected by pricking the thorax with a needle (0.01 mm, Minutien pins, Fine Science Tools, CA) dipped in a suspension (OD_600_ 0.4) of coevolving *P. entomophila* pathogen. The infected flies were transferred to a Plexiglas cage (14cm length × 16cm width × 13cm height) and provided with a fresh food plate which was replaced on alternate days. A significant number of flies died within 24-48h post infection. The number of dead flies in the cage was counted and 10-15 dead flies per sex were collected and stored at 4°C for further use. These flies were later used to extract the bacteria to infect the next generation. Host mortality was recorded till four days post infection as after that time point, host mortality is not found to be significant (Gupta, Ali, & Prasad, 2013). Around 200 individuals survived the infection and they would contribute to the next generation. 96 h post infection, the surviving flies were provided with a fresh food plate for oviposition for 18h. After that, the eggs were collected into 10 food vials at a density of 70 eggs per vial to start the next generation.

Preparation and isolation of coevolved pathogen

We collected 10-15 dead flies of each sex during the peak mortality period post infection. These dead flies were used to isolate the pathogen to infect the next generation of hosts. Out of the collected dead flies, five flies were randomly picked and were washed in absolute ethanol for surface-sterilization. These flies were randomly transferred to micro-centrifuge tubes at a density of five per tube. These flies were then crushed in 10mM MgSO_4_ using a homogenizer and a pestle and were serially diluted 3-4 times. This diluted sample was plated (100µl) on LB agar plates containing ampicillin and these plates were incubated overnight at 27°C. Next day, these plates were observed for colonies and were stored to infect next generation flies. We randomly picked 11-12 colonies from different regions of the plate and inoculated an overnight culture one night before the bacterial infection. This protocol was repeated every generation to isolate evolved bacteria from the host, to infect the next host generation.

It should be noted that the bacteria isolated from a given population of hosts were used to infect hosts of the same population in the next generation. For example, bacteria isolated from dead flies of Coev 1 would be used to infect hosts from Coev 1 in the next generation and not to any other host i.e. Coev 2, Coev 3 and Coev 4. This was also applicable to rest of the coevolving pathogens and hosts i.e., Coev 2, 3 and 4. Since there were four different coevolving *Pseudomonas entomophila* pathogens, each coevolving pathogen was labeled as ‘B1Pe’, ‘B2Pe’, ‘B3Pe’ and ‘B4Pe’ respectively.

As the generation time of a pathogen is shorter relative to the host, it resulted in rapid evolution of the coevolving pathogen as compared to the coevolving host. This was evident from the fact that we observed increased mortality of the coevolving host when we infected the host with the new coevolution generation of the pathogen. Hence, to provide sufficient time to the host to (co)evolve and to maintain good breeding adult numbers, we started to infect two consecutive host generations with one generation of pathogen after the 5^th^ generation cycle i.e. after two host generations of evolution, a new coevolution cycle for host and pathogen was proceeded. In other words, a fresh sample of coevolved bacteria was isolated from the host only after allowing it to evolve for two generations against the coevolved bacteria from the previous generation. This practice ensured sufficient time for the coevolving host to coevolve with the pathogen.

**2. Adapt (host adaptation against non-evolving pathogen):** In Adapt selection regime, there were four populations labelled as Adapt (1-4). From each population, eggs were collected in 10 fresh food vials at a density of 70 eggs per vial. Each food vial contained 6-7ml banana-jaggery fly media and these vials were incubated at the standard conditions as mentioned in the Coev regime. These flies start to eclose on 9^th^ day and by 10^th^ day, peak eclosion of flies is attained. On 12^th^ day post egg collection, when these flies were 2-3 days old , 150 males and 150 females (15 males and 15 females from each vial) from each of the rearing vial were randomly chosen. These flies were anesthetised by using CO_2_ and were infected with the *P. entomophila* pathogen, as mentioned above at an OD_600_ 0.5. Flies from this regime were infected with a non-evolving or non-changing *P. entomophila* pathogen i.e. only the host evolved in response to the non-changing or static pathogen. Post-infection, flies were transferred to the cage and were provided with a fresh food plate which was replaced on alternate day. We monitored the host mortality for four days post infection. After 96h post infection, no significant fly mortality occurs. After this time, around 200 individuals were left in the cage and were provided with a fresh food plate for oviposition for 18h. Later, eggs were collected in 10 fresh food vials at a density of 70 eggs per vial to start the next generation.

Please note that this pathogen was taken from the ancestral bacterial stock every generation and this is the same pathogen which was used to derive the coevolving *P. entomophila*. Therefore, this pathogen would be designated as ‘Ancestral Pe’ or ‘Anc Pe’.

**Co.S (Sham infection Control):** From each of the four populations of Co.S selection regime (Co.S(1-4)) eggs were collected in 10 food vials containing 6-7ml banana-jaggery fly media, at a density of 70 eggs per vial. These vials were later incubated under standard laboratory conditions as mentioned above. On 12^th^ day post egg collection, when these flies had eclosed and were 2-3 days old,100 males and 100 females (10males and 10 females from each vial) from each population were randomly chosen. These flies were anesthetised under CO_2_ and were pricked by a needle dipped in sterile 10mM MgSO_4_ solution. This regime was used as an infection/pricking control as it caused just 0-1% mortality in flies. Post treatment, these flies were transferred to Plexiglas cages and were provided with fresh food plates which were replaced with fresh food plates on alternate days. After 96h, a fresh food plate was provided for oviposition for 18h. Eggs laid during this window were collected in 10 fresh food vials at a density of 70 eggs per vial, to start the next generation.

**Co.U (Unhandled Control):** The four populations within Co.U selection regime were labelled as Co.U(1-4). From each population, eggs were collected in 10 food vials containing 6-7ml banana-jaggery food, at a density of 70 eggs per vial. On 12^th^ day post egg collection, from each population, 100 males and 100 females (10 males and 10 females from each vial) were randomly sorted as males and females, under light CO_2_ anaesthesia. These flies were neither infected nor pricked with needle. Post treatment, flies were transferred to the cage and were provided with a fresh food plate which was replaced with a fresh food plate on alternate days. After 96h, a fresh food plate was provided for oviposition for 18h. After that, obtained eggs were collected in 10 fresh food vials at a density of 70 eggs per vial.

**Bacterial Infection for experiments**

The glycerol stock of the bacteria stored at -80°C, was used to set up an overnight primary culture in a conical flask containing Luria Bertani (LB) medium. The next morning, this overgrown primary culture was used to start the secondary bacterial culture in fresh LB. This was allowed to grow for 3-4 hours. Afterwards, the final suspension with the desired OD was prepared from the secondary culture by dissolving the bacterial pellet in sterile 10 mM MgSO_4_ solution. This bacterial slurry was used to infect the flies.

In order to infect flies, they were pricked on the thorax under mild CO_2_anaesthesia using a fine minutein needle 0.01mm (Fine Science Tools, CA) dipped in the bacterial suspension (or MgSO_4_ solution for sham infections. All the bacterial infections in the experimental flies from each population were done at an OD 0.44.

**Block design**

As mentioned above we used four of the BRB(1-4)populations as ancestral populations to derive our selection regimes. From each BRB population, we derived one replicate each for the four selection regimes. For example, from BRB 1, we derived Coev 1, Adapt 1, Co.S 1 and Co.U 1; from BRB 2, we derived Coev 2, Adapt 2, Co.S 2 and Co.U 2 populations and so on. Thus, populations with common subscripts shared a common ancestry and were hence more closely related to each other compared to populations with different subscripts. For example, Coev 1, Adapt 1, Co.S 1 and Co.U 1 (all derived from BRB 1) were more closely related to each other than any of them were to Coev 2, etc. Additionally, populations from one block were always handled together during stock maintenance and during experimentation. Therefore, populations with common subscripts were treated as statistical blocks. The sixteen populations used in this study were grouped into four distinct blocks. For example, Coev 1, Adapt 1, Co.S 1 and Co.U 1 formed block 1 and so on.

**Standardisation of fly populations**

To observe the patterns of adaptations in the coevolving populations, local adaptation experiment was conducted after 19 cycles of host-pathogen coevolution. Before starting the experiment, flies from each regime were standardized (Rose, 1984) to account for non-genetic parental effects that might have affected the traits under study. To conduct the experiments, we took a large subsample from the stock populations. All experiments were done on this subsample. We collected eggs from the stock populations and dispensed the eggs at a density of 70 eggs per vial in 7 food vials containing 6-7m banana-jaggery fly media. On the 12^th^ day post egg collection, when these flies were roughly 2-3 days old, were transferred into cages, and were not subjected to any selection. Each cage contained approximated 500 flies and was supplied with a food plate and were incubated. The food plate was smeared with live yeast paste for forty eight hours to boost fecundity. After 48 hours, a fresh food plate was given in each cage for egg laying. After eighteen hours, eggs were collected from these plates at a density of seventy eggs per vial, with forty vials being collected from each selection regime by block combination. These vials were incubated under standard laboratory conditions of 25ºC temperature, 50-60% relative humidity and under 12:12LD cycle. These flies were later used for experimentation.

**Results –**

**Figure S1:** Mean fecundity of females from each of the coevolving populations post infection by sympatric or allopatric coevolving pathogens, or post sham infection. Error bars represent standard errors. Data for each of the four coevolving host populations is plotted in separate graphs with (a), (b), (c) and (d) representing Coev 1 hosts, Coev 2 hosts, Coev 3 hosts and Coev 4 hosts respectively. H1, H2, H3 and H4 represent Coev 1, Coev 2, Coev 3 and Coev 4, and P1, P2, P3 and P4 represent coevolving pathogens from those populations, i.e., B1Pe (coevolving Pe from block 1), B2Pe (coevolving Pe from block 2), B3Pe (coevolving Pe from block 3) and B4Pe (coevolving Pe from block 4) respectively. Within each graph, pink, grey, light blue, orange and purple colours represent fecundity of female hosts post infection by B1Pe, B2Pe, B3Pe, B4Pe and sham infection respectively.

(a) (b)


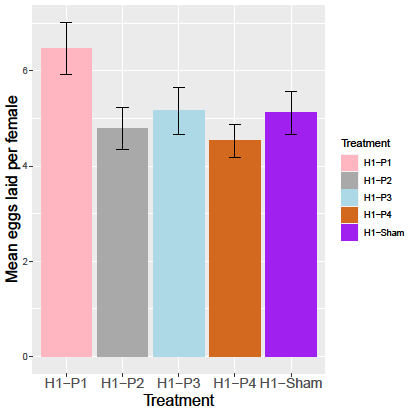

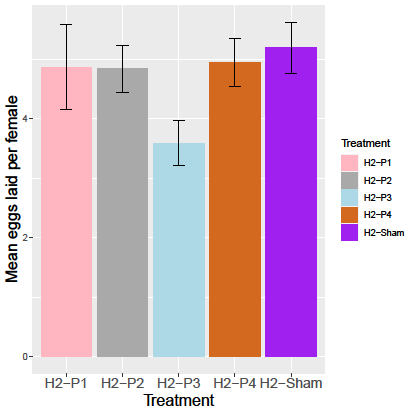


(c) (d)


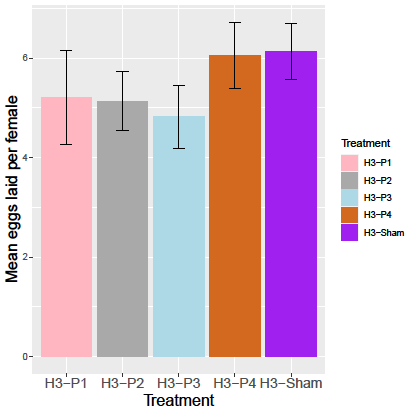

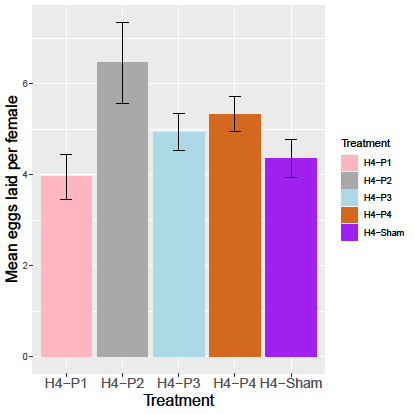


**Table S1:** Summary of mixed model anova for fecundity of females from the four coevolving populations when subjected to five different infections treatments: sham infection or infected with B1Pe, B2Pe, B3Pe or B4Pe. Treatment was considered as a fixed factor while experimental replicate was considered as a random factor.

| **Coev 1 (Host 1)** | | | | | | |
| --- | --- | --- | --- | --- | --- | --- |
| **Fixed Coefficients** | **Sum sq** | **Mean sq** | **Num Df** | **Den Df** | **F value** | **P(>F)** |
| Treatment | 31.314 | 7.828 | 4 | 62.835 | 2.678 | **0.0396*** |
| **Random Effects** | **npar** | **logLik** | **ARC** | **LRT** | **Df** | **P(>chisq)** |
| Replicate | 6 | -134.6 | 281.20 | 0.4785 | 1 | 0.489 |
|  |  |  |  |  |  |  |
| **Coev 2 (Host 2)** | | | | | | |
| **Fixed Coefficients** | **Sum sq** | **Mean sq** | **Num Df** | **Den Df** | **F value** | **P(>F)** |
| Treatment | 22.084 | 5.5209 | 4 | 62.787 | 1.7255 | 0.1555 |
| **Random Effects** | **npar** | **logLik** | **ARC** | **LRT** | **Df** | **P(>chisq)** |
| Replicate | 6 | -137.23 | 286.47 | 0.2339 | 1 | 0.6286 |
| **Coev 3 (Host 3)** | | | | | | |
| **Fixed Coefficients** | **Sum sq** | **Mean sq** | **Num Df** | **Den Df** | **F value** | **P(>F)** |
| Treatment | 19.285 | 4.821 | 4 | 65 | .7164 | 0.5838 |
| **Random Effects** | **npar** | **logLik** | **ARC** | **LRT** | **Df** | **P(>chisq)** |
| Replicate | 6 | -160.79 | 333.58 | -1.13e-13 | 1 | 1 |
|  |  |  |  |  |  |  |
| **Coev 4 (Host 4)** | | | | | | |
| **Fixed Coefficients** | **Sum sq** | **Mean sq** | **Num Df** | **Den Df** | **F value** | **P(>F)** |
| Treatment | 52.731 | 13.183 | 4 | 62.934 | 3.358 | **0.0143*** |
| **Random Effects** | **npar** | **logLik** | **ARC** | **LRT** | **Df** | **P(>chisq)** |
| Replicate | 6 | -145.97 | 303.94 | 2.647 | 1 | 0.1037 |

**Table S2 :** The output of Cox’s proportional hazards models for different Coev hosts post infection with their sympatric and allopatric pathogens. Hazard rates are expressed relative to the hazard rates of the default level of each fixed factor, which are constrained to be 1. The default level for “Pathogen” is Pe 1, while the default level for “Sex” is Females (F). Lower CI and Upper CI indicate lower and upper bounds of 95% confidence intervals. Confidence intervals that do not contain 1 signify statistical significance and are shown in bold. Higher hazard rates are equivalent to lower survivorship in the hosts. In the table, B1Pe, B2Pe, B3Pe and B4Pe represent coevolving Pe from population Coev 1, Coev 2, Coev 3 and Coev 4.

| **Coev 1 (Host 1)** | | | |
| --- | --- | --- | --- |
| **Fixed Coefficients** | **Hazard rate** | **Lower CI** | **Upper CI** |
| PathogenB2Pe | 1.8922 | **1.2808** | **2.7951** |
| PathogenB3Pe | 0.27869 | **0.1459** | **0.5321** |
| PathogenB4Pe | 0.44907 | **0.2596** | **0.7768** |
| SexM | 1.00655 | 0.6468 | 1.566 |
| PathogenB2Pe:SexM | 0.83150 | 0.4735 | 1.4599 |
| PathogenB3Pe:SexM | 1.79840 | 0.7791 | 4.1507 |
| PathogenB4Pe:SexM | 1.00878 | 0.4646 | 2.1899 |
| **Random effects** | | | |
| **Group** | | **Variance** | |
| Population/Bacteria/Sex | | 0.0003871122 | |
| Population/Bacteria | | 0.0003944583 | |
| Population | | 0.0465781883 | |

**(b)**

| **Coev 2 (Host 2)** | | | |
| --- | --- | --- | --- |
| **Fixed Coefficients** | **Hazard rate** | **Lower CI** | **Upper CI** |
| PathogenB2Pe | 0.86923 | 0.569726 | 1.326309 |
| PathogenB3Pe | 0.57549 | **0.359263** | **0.921917** |
| PathogenB4Pe | 0.48734 | **0.297126** | **0.799395** |
| SexM | 2.90052 | **2.052585** | **4.098414** |
| PathogenB2Pe:SexM | 0.28692 | **0.162042** | **0.508088** |
| PathogenB3Pe:SexM | 0.83671 | 0.475352 | 1.472851 |
| PathogenB4Pe:SexM | 0.61044 | 0.330714 | 1.126821 |
| **Random effects** | | | |
| **Group** | | **Variance** | |
| Population/Bacteria/Sex | | 8.7399e-05 | |
| Population/Bacteria | | 4.0892e-04 | |
| Population | | 6.1896e-02 | |

**(c)**

| **Coev 3 (Host 3)** | | | |
| --- | --- | --- | --- |
| **Fixed Coefficients** | **Hazard rate** | **Lower CI** | **Upper CI** |
| PathogenB2Pe | 1.182348 | 0.868142 | 1.610267 |
| PathogenB3Pe | 0.286201 | **0.1821** | **0.449868** |
| PathogenB4Pe | 0.74892 | 0.534566 | 1.049276 |
| SexM | 1.86363 | 1.391942 | 2.495028 |
| PathogenB2Pe:SexM | 0.705816 | 0.468415 | 1.063537 |
| PathogenB3Pe:SexM | 1.19519 | 0.683315 | 2.090493 |
| PathogenB4Pe:SexM | 0.42542 | 0.26387 | 0.685985 |
| **Random effects** | | | |
| **Group** | | **Variance** | |
| Population/Bacteria/Sex | | 2.780e-05 | |
| Population/Bacteria | | 5.0921e-05 | |
| Population | | 1.316e-01 | |

**(d)**

| **Coev 4 (Host 4)** | | | |
| --- | --- | --- | --- |
| **Fixed Coefficients** | **Hazard rate** | **Lower CI** | **Upper CI** |
| PathogenB2Pe | 1.28086 | 0.98167 | 1.671297 |
| PathogenB3Pe | 0.45907 | **0.330549** | **0.637628** |
| PathogenB4Pe | 0.30259 | **0.208754** | **0.438673** |
| SexM | 1.79395 | **1.388189** | **2.317989** |
| PathogenB2Pe:SexM | 0.65156 | **0.456074** | **0.930996** |
| PathogenB3Pe:SexM | 1.27186 | 0.841558 | 1.922257 |
| PathogenB4Pe:SexM | 0.52763 | **0.313549** | **0.887985** |
| **Random effects** | | | |
| **Group** | | **Variance** | |
| Pathogen/Bacteria/Sex | | 8.177e-06 | |
| Pathogen/Bacteria | | 1.1349e-05 | |
| Pathogen | | 0.238 | |

**Table S3:** The output of Cox’s proportional hazards models for each of the coevolving pathogens from different populations when they infect their sympatric as well as allopatric hosts. Hazard rates are expressed relative to the hazard rates of the default level of each fixed factor, which are constrained to be 1. The default level for “Host” is Coev 1, while the default level for “Sex” is Female (F). Lower CI and Upper CI indicate lower and upper bounds of 95% confidence intervals. Confidence intervals that do not contain 1 signify statistical significance and are shown in bold. Higher hazard rates are equivalent to lower survivorship in the hosts.

| **Pathogen-1** | | | |
| --- | --- | --- | --- |
| **Fixed Coefficients** | **Hazard rate** | **Lower CI** | **Upper CI** |
| Coev2 | 1.1468 | 0.754802 | 1.742464 |
| Coev3 | 2.0170 | **1.383477** | **2.940266** |
| Coev4 | 3.0914 | **2.153081** | **4.438427** |
| SexM | 0.9565 | 0.618598 | 1.47905 |
| Coev2:SexM | 3.0360 | **1.740896** | **5.294137** |
| Coev3:SexM | 1.9420 | **1.149469** | **3.280842** |
| Coev4:SexM | 1.8735 | **1.129867** | **3.10651** |
| **Random effects** | | | |
| **Group** | | **Variance** | |
| Pathogen/Host/Sex | | 8.481e-06 | |
| Pathogen/Host | | 1.2436e-05 | |
| Pathogen | | 1.9507e-01 | |

**2(b) Pathogen 2**

| **Pathogen-2** | | | |
| --- | --- | --- | --- |
| **Fixed Coefficients** | **Hazard rate** | **Lower CI** | **Upper CI** |
| Coev2 | 0.5576 | **0.379614** | **0.819468** |
| Coev3 | 1.3395 | 0.977947 | 1.834735 |
| Coev4 | 2.2707 | **1.693674** | **3.044084** |
| SexM | 1.1475 | 0.830274 | 1.585817 |
| Coev2:SexM | 0.7275 | 0.416404 | 1.271249 |
| Coev3:SexM | 1.1614 | 0.752993 | 1.791405 |
| Coev4:SexM | 1.0300 | 0.684956 | 1.548985 |
| **Random effects** | | | |
| **Group** | | **Variance** | |
| Population/Host/Sex | | 1.2099e-06 | |
| Population/Host | | 6.4709e-05 | |
| Population | | 1.6929e-01 | |

**2(c) Pathogen 3**

| **Pathogen-3** | | | |
| --- | --- | --- | --- |
| **Fixed Coefficients** | **Hazard rate** | **Lower CI** | **Upper CI** |
| Coev2 | 2.6841 | **1.335626** | **5.39353** |
| Coev3 | 2.3652 | **1.163346** | **4.808571** |
| Coev4 | 5.8265 | **3.051093** | **11.12617** |
| SexM | 1.9716 | 0.950564 | 4.089816 |
| Coev2:SexM | 1.2389 | 0.526397 | 2.915963 |
| Coev3:SexM | 1.1350 | 0.474687 | 2.714207 |
| Coev4:SexM | 1.1647 | 0.524033 | 2.588556 |
| **Random effects** | | | |
| **Group** | | **Variance** | |
| Population/Host/Sex | | 6.5928e-05 | |
| Population/Host | | 1.5944e-04 | |
| Population | | 6.8622e-02 | |

**2(d) Pathogen 4**

| **Pathogen-4** | | | |
| --- | --- | --- | --- |
| **Fixed Coefficients** | **Hazard rate** | **Lower CI** | **Upper CI** |
| Coev2 | 1.2904 | 0.706876 | 2.355846 |
| Coev3 | 3.5135 | **2.099082** | **5.880254** |
| Coev4 | 2.1811 | **1.260363** | **3.774244** |
| SexM | 1.0130 | 0.536333 | 1.913244 |
| Coev2:SexM | 1.8454 | 0.821026 | 4.147891 |
| Coev3:SexM | 0.7826 | 0.373477 | 1.64017 |
| Coev4:SexM | 0.9227 | 0.422697 | 2.013753 |
| **Random effects** | | | |
| **Group** | | **Variance** | |
| Population/Host/Sex | | 1.0926e-05 | |
| Population/Host | | 1.4449e-05 | |
| Population | | 1.6656e-02 | |
